# Leishmaniasis and Tularemia Coinfection: A Case Report of Neglected Causes of Cervical Lymphadenopathy

**DOI:** 10.7759/cureus.64605

**Published:** 2024-07-15

**Authors:** Mark Grigoryan, Violeta Manukyan, Tatevik Grigoryan, Hripsime Apresyan

**Affiliations:** 1 Infectious Diseases, Muratsan University Hospital Complex, Yerevan, ARM; 2 Infectious Diseases, Yerevan State Medical University, Yerevan, ARM

**Keywords:** visceral leishmaniasis (vl), meglumine antimoniate, cervical lymphadenopathy, tularemia, localized leishmanial lymphadenopathy

## Abstract

Leishmania and tularemia are infectious diseases that both can present with lymphadenopathy. Leishmania typically causes visceral or cutaneous forms, while tularemia can result in glandular tularemia characterized by lymphadenitis. We report a case of a patient presenting with localized cervical lymphadenopathy diagnosed with both leishmaniasis and tularemia. This case underscores the importance of considering both pathogenic agents in the differential diagnosis of localized lymphadenitis. Early treatment is crucial to prevent the dissemination of these infections.

## Introduction

Cervical lymphadenopathy is defined as the presence of one or more lymph nodes of more than 1 cm in diameter, with or without an abnormality in character. The etiology of cervical lymphadenopathy is categorized into four main groups: infectious, immunologic, malignancy, and miscellaneous [1]. Infectious causes include not only viral (herpes viruses, adenovirus, hepatitis B virus, etc) but bacterial (group A streptococcal disease, brucellosis, tularemia, leptospirosis), spirochetal (syphilis, Lyme disease), parasitic (toxoplasmosis, leishmaniasis, malaria) and fungal (blastomycosis, histoplasmosis, coccidioidomycosis) diseases [2].

Francisella tularensis, an aerobic and fastidious gram-negative bacterium, is a cause of tularemia, a zoonotic infection. An infection in humans occurs after contact with infected animals or invertebrates [3]. The clinical manifestations may range from asymptomatic disease to septic shock [3]. Similarly to tularemia, visceral leishmaniasis can manifest with fever, fatigue, and lymphadenopathy. It is a vector-borne disease transmitted by phlebotomine sandflies. The etiologic agent in Armenia is *Leishmania infantum* [4]. 

Most common cervical lymphadenopathy in children is self-limited and does not require treatment [5]. Antibacterial therapy should be initiated promptly for patients suspected or confirmed to have tularemia. Although self-limitation of infection with no specific treatment has been reported, early effective treatment leads to less morbidity. Spontaneous resolution of visceral leishmaniasis is rare. Nevertheless, some self-limited cases have been recorded in a cohort of Brazilian children [6].

## Case presentation

Twenty days after the onset of clinical symptoms, a 12-year-old boy was brought to Muratsan University Hospital Complex with complaints of fever, malaise, loss of appetite, and cervical lymphadenopathy.

The patient was well-nourished. All vaccines were up-to-date. He had no known allergies. Epidemiologic history revealed that the patient lived in an endemic region for visceral leishmaniasis and had contact with a severe acute respiratory syndrome-coronavirus-2 (SARS-CoV-2)-positive patient.

The current disease manifested acutely with cervical lymph node enlargement followed by fever three to four days later. The primary care pediatrician consulted an oral and maxillofacial surgeon who prescribed amoxicillin/clavulanate, but it did not improve the patient's condition. During a subsequent examination, positive antileishmanial antibodies were found in the patient's serum, and the patient was then referred to Muratsan University Hospital Complex.

During the presentation, the boy seemed unwell, although he was afebrile. He had a pale complexion, and there were no visible rashes on his skin or mucous membranes. Physical examination showed enlarged, non-tender, firm, and mobile submandibular lymph nodes on both sides of his neck. Other tests conducted on his body systems did not show any abnormalities. The complete blood count, biochemical profile, and urinalysis results were all within the reference ranges (Table 1).

**Table 1:** Laboratory investigation results WBC - white blood cells; RBC - red blood cells; HGB - hemoglobin concentration; MCV - mean cell volume; MCH - mean cell hemoglobin; MCHC - mean cell hemoglobin concentration; PLT - platelets; CRP - C-reactive protein; APTT - activated partial thromboplastin time; PT - prothrombin time; INR - international normalized ratio

Test	At the time of admission	On the eighth day of hospitalization	Reference	Unit
WBC	4.73	5.11	4–10	10^9^ /L
Lymphocyte	2.12	2.32	1.0–3	10^9^ /L
Neutrophil	1.98	2.16	1.8–6.4	10^9^ /L
RBC	5.03	4.92	3.5–5.5	10^12^ /L
HGB	125	121	110–160	g/L
MCV	75.7	75.5	82–95	Fl
MCH	24.9	24.5	27–31	Pg
MCHC	329	324	320–360	g/L
PLT	210	177	100–300	10^9^ /L
CRP	1.80	1.08	0–10	mg/L
Total protein	70.7	-	65-85	g/L
Albumin	40.7	-	39-52	g/L
Creatinine	40	-	27-62	µmol/L
Total bilirubin	6.5	-	2.55-20.5	µmol/L
Indirect bilirubin	3.6	-	0-5.1	µmol/L
Direct bilirubin	2.9	-	2.55-15.4	µmol/L
Alkaline Phosphatase	126	-	20-147	IU/L
Glucose	5.36	-	4.2-6.4	mmol/L
Ferritin	25.45	-	14-124	Ng/mL
Alanine aminotransferase (ALT)	10.4	-	<40	U/L
Aspartate aminotransferase (AST)	18.1	-	<35	U/L
gamma-glutamyltransferase (GGT)	11.2	-	7-32	U/L
APTT	-	33.1	28–44.5	Second
PT	-	14.5	11–14.5	Second
INR	-	1.26	1.0-2.0	
Fibrinogen	-	2.21	2.0–4	g/L
Na+	140.6	-	135-155	mmol/L
K+	3.55	-	3.1-5.2	mmol/L

**Figure 1:**
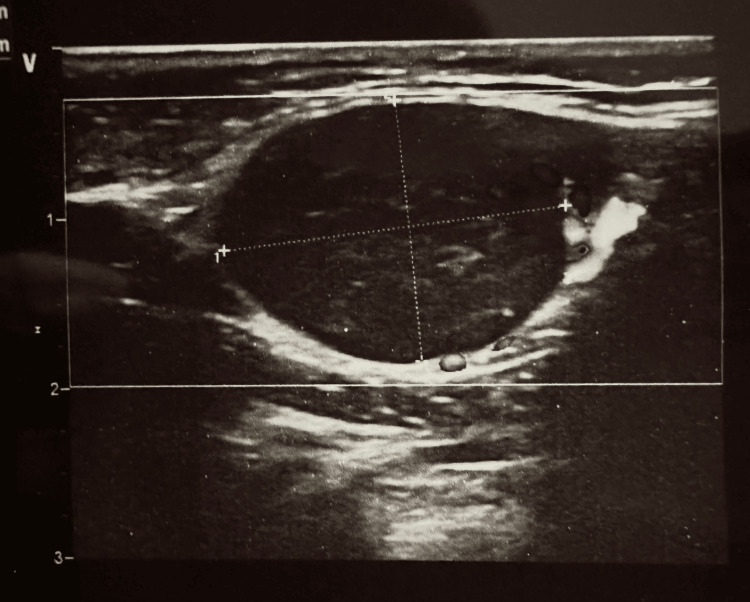
Ultrasonography revealed bilaterally enlarged cervical lymph nodes, with the largest up to 2.4x0.8 cm in size and a length-to-width ratio of less than 0.5

Atypical mononuclear cells were absent on the blood smear. The following tests, including hepatitis B surface antigen (HBsAg), anti-human immunodeficiency virus (HIV)-1/2 and anti-hepatitis C virus (HCV) antibodies, Epstein Barr virus (EBV) IgM, cytomegalovirus (CMV) IgM, as well as toxoplasma IgM, were negative (Table 2). Serologic examinations for brucellosis and tularemia were pending. The chest X-ray showed a cardiothoracic index of 0.51 (normal range <0.5). The lung ventilation was normal. The abdominal ultrasound scan was unremarkable, except for mild splenomegaly (13.8x5.1 cm). The ultrasonography of lymph nodes revealed that the largest axillary lymph nodes measured up to 1.3x0.6 cm in size, with a length-to-width ratio of less than 0.5. The cervical lymph nodes were bilaterally enlarged, with the largest up to 2.4x0.8 cm in size and a length-to-width ratio of less than 0.5 (Figure 1). Additionally, there was a nonhomogeneous, hypoechoic lesion measuring up to 2.1x1.4 cm in size and a length-to-width ratio greater than 0.5, located in the submandibular area.

**Table 2:** Laboratory tests for infections EBV - Epstein Barr virus; CMV - cytomegalovirus; HBsAg - hepatitis B surface antigen; anti-HCV antibodies - anti-hepatitis C antibodies; anti-HIV_1/2_ antibodies - anti-human immunodeficiency virus -1/2 antibodies; PCR - polymerase chain reaction

Infectious agent	Test	Result
Viral	EBV IgM	Negative
	CMV IgM	Negative
	HBsAg	Negative
	Anti-HCV antibodies	Negative
	Anti-HIV_1/2_ antibodies	Negative
	SARS-CoV-2 PCR	Negative
Bacterial	anti-Brucella antibodies	Negative
	Francisella tularensis IgM, IgG (on the day of admission)	Positive
	Francisella tularensis IgM, IgG (on the 10^th^ day of admission)	Positive
Parasitic	Toxoplasma IgM	Negative
	Antileishmanial antibodies	Positive

A primary diagnosis of visceral leishmaniasis, localized unspecified lymphadenopathy was made, pending confirmation. The patient was treated with a combination of intravenous ceftriaxone 50 mg/kg/day and metronidazole 25 mg/kg/day to cover the most common pathogens causing localized lymphadenopathy, including gram-positive cocci and anaerobe microorganisms.

The following day, the patient's serology tests for tularemia were positive (IgM-1.5 U/L, IgG-2.0 U/L), and brucellosis was negative. The treatment strategy was changed to intravenous ciprofloxacin 10mg/kg twice a day to address glandular tularemia.

**Figure 2:**
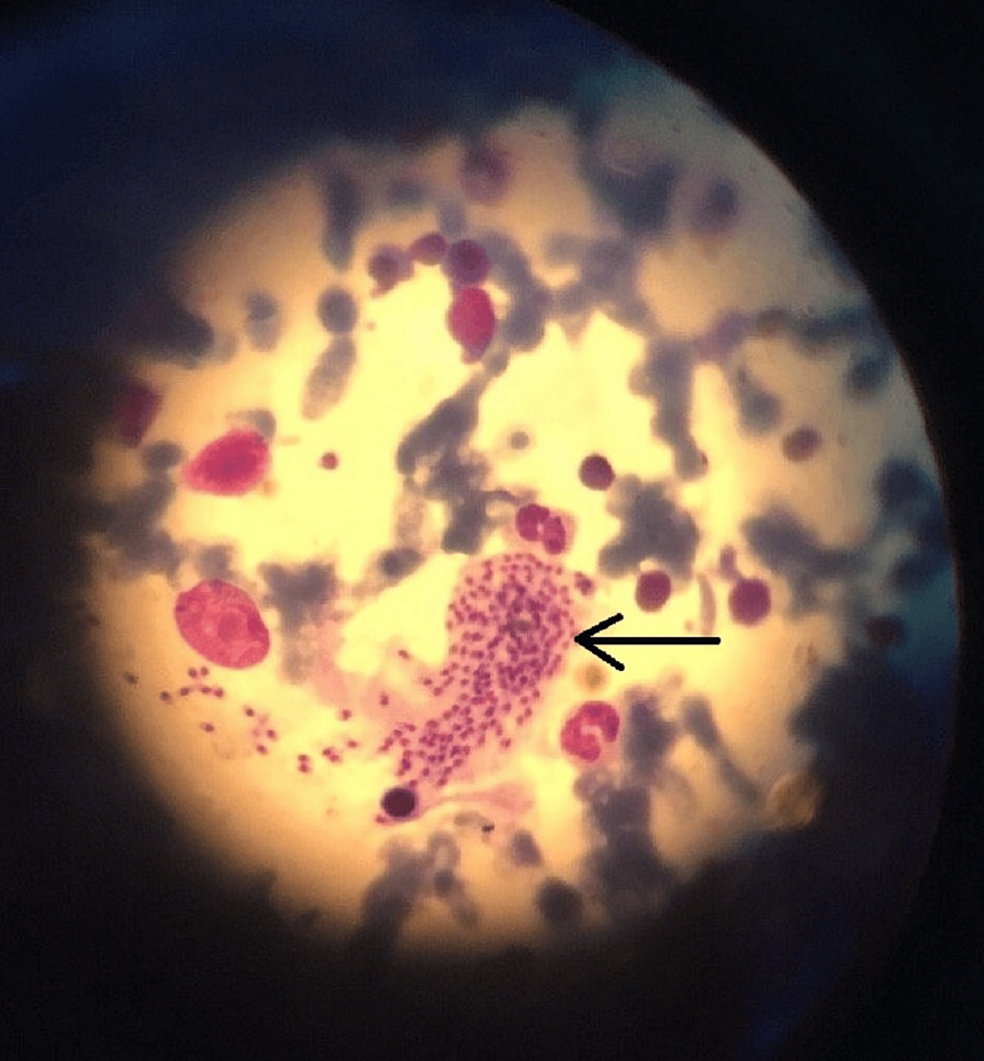
Leishmania amastigotes on lymph node aspirate (100x magnification)

Although the ultrasound scan showed no remarkable changes, the complete blood count and biochemistry profile remained within normal ranges after seven days of treatment (Table 1). The second blood sample was obtained to measure the level of antibodies against tularemia, which showed more than a four-fold increase in immunoglobulin titers. However, after 14 days of treatment with no improvement, the patient was followed up for another five days. On day 12, fine needle aspiration of the submandibular lymph node was performed and sent for microscopic examination. The microscopic examination revealed granulomatous disease more consistent with leishmaniasis. The diagnosis was confirmed by detecting *Leishmania *amastigotes on histology (Figure 2).

**Figure 3:**
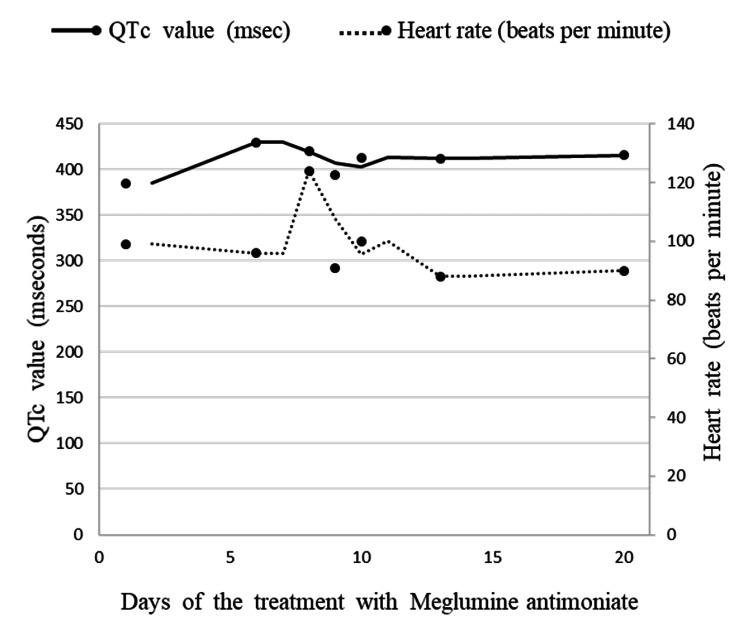
ECG monitoring - heart rate and QTc values during the treatment course with meglumine antimoniate ECG - electrocardiogram; QTc - corrected QT interval; msec - deci second

Therefore, the patient was diagnosed with localized leishmanial lymphadenopathy since there was no indication of involvement in other reticuloendothelial organs (e.g., hepatosplenomegaly or pancytopenia). Meglumine antimoniate of 75 mg/kg/day intramuscularly has been administered. Routine checkups for cardiac (electrocardiogram, echocardiography), liver (alanine aminotransferase, aspartate aminotransferase, total protein, albumin, prothrombin time, activated partial thromboplastin time), pancreatic (alpha- and pancreatic amylases, glucose), and renal (creatinine, electrolytes) function tests were performed. The baseline test results were normal. An electrocardiogram revealed sinus arrhythmia, a heart rate of 99 beats per minute, and a corrected QT interval (QTc) of 385 msec (deci seconds). Consequently, echocardiography was performed, and there were no contraindications for the treatment with antileishmanial drugs. Considering the toxic effects of meglumine antimoniate, cardiac, liver, and renal functions were being monitored during the treatment course. There was no adverse effect of the drug according to monitoring data (Figure 3).

On the 15th day of antileishmanial treatment, the patient spiked a fever of 38.5^0^C. The injection site was tender, and the skin was warm and red. Intravenous followed by oral amoxicillin/clavulanate 1.2 g twice a day was administered to treat the injection site inflammation. Three days after the onset of the treatment, the patient was afebrile. An abdominal ultrasound scan revealed no hepatosplenomegaly. Moreover, the cervical lymph nodes on ultrasound examination had a reduction in size after the treatment.

The boy was discharged with clinical and laboratory improvement. The length of hospital stay was 42 days.

## Discussion

We reported a noteworthy case of coinfection of two rare pathogens. While we initially found antileishmanial antibodies in the patient's blood, it was unreliable in diagnosing visceral leishmaniasis.

Approximately 95% of the Armenian population is endemic to tularemia [7]. Therefore, we test patients for tularemia along with common viral and parasitic infections that cause lymphadenopathy (e.g., EBV, CMV, *Toxoplasma spp*, etc.). Patients presenting with acute illness should be managed based on clinical suspicion. Tularemia is generally diagnosed through serological testing, which involves identifying a fourfold or greater increase in antibody titers against *Francisella* *tularensis* between acute and convalescent serum samples. Serologic tests for tularemia can cross-react with heterophile antibodies and antibodies to other gram-negative bacteria like *Brucella *or *Legionella*; however, these cross-reactions usually result in low, non-diagnostic titers [3]. The patient was diagnosed with tularemia due to a fourfold increase in antibody titers and received appropriate antimicrobial treatment. However, the enlarged lymph nodes did not decrease in size, so we continued the patient's evaluation for leishmaniasis. While initial testing detected antileishmanial antibodies in the patient's blood, this finding alone was insufficient for diagnosing visceral leishmaniasis in the absence of clinical manifestations such as hepatosplenomegaly and pancytopenia.

Visceral leishmaniasis is a re-emerging disease in Armenia. Its most common symptoms are malaise, fever, weight loss, and splenomegaly (with or without hepatomegaly) over weeks to months [8]. However, our patient did not show signs of reticuloendothelial system disease (i.e., liver, spleen, and bone marrow are spared), except for lymphadenopathy, which made diagnosing visceral leishmaniasis challenging. As per guidelines, we performed a cervical lymph node biopsy only after four weeks of treatment when we saw no significant response (Figure 3). 

**Figure 4:**
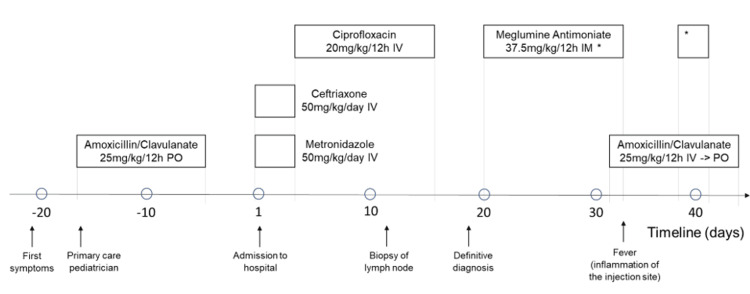
Drugs administered to the patient before and after admission to the hospital PO - per os; IV - intravenous; IM - intramuscular * Meglumine antimoniate 37.5 mg/kg/12h intramuscular administration resumed

Excisional biopsy is currently considered the best method of diagnosis. It is recommended to perform an early biopsy for children who are over 10 years old, have lymph nodes larger than 2 cm in size, and have multiple sites of adenopathy or supraclavicular nodes, as they may be at higher risk for malignancy [9].

Due to fine needle aspiration of the lymph node, a rare diagnosis was confirmed: localized leishmanial lymphadenopathy. There are a few cases of localized leishmanial lymphadenopathy described in the literature during a large outbreak in Spain [10]. The vast majority of the disease is typically observed in immunocompetent patients. However, localized disease does have the potential to progress to a systemic illness, and as a result, it is preferable to focus on the management of visceral leishmaniasis. The first line treatment of visceral leishmaniasis is liposomal amphotericin B. The FDA-approved regimen for immunocompetent patients consists of 3 mg per kg daily, by IV infusion, on days one to five, 14, and 21 (total dose of 21 mg/kg). We had to administer the second-line treatment with meglumine antimoniate due to the unavailability of the first-line treatment drug in our country at that time.

## Conclusions

In summary, two uncommon causative agents can present with the same clinical symptoms. Conducting comprehensive patient evaluations may help reduce the possibility of misdiagnosing mixed infections. The failure of empirical treatment for tularemia, combined with positive antileishmanial antibody titers, highlighted the need to utilize additional diagnostic tools. An excisional biopsy, considered the most accurate diagnostic technique, is recommended early for children older than 10 years who present with lymph nodes larger than 2 cm, involvement of multiple sites of adenopathy, or supraclavicular nodes, as these conditions may indicate an increased risk of malignancy. Practitioners should consider *Leishmania spp.* as a potential cause of regional lymphadenopathy and include leishmaniasis in their differential diagnosis for chronic or persistent lymph node enlargement.
